# Deciphering olfactory receptor binding mechanisms: a structural and dynamic perspective on olfactory receptors

**DOI:** 10.3389/fmolb.2024.1498796

**Published:** 2025-01-08

**Authors:** Jingtao Wang, Qidong Zhang, Wu Fan, Qingzhao Shi, Jian Mao, Jianping Xie, Guobi Chai, Chenglei Zhang

**Affiliations:** ^1^ College of Chemistry, Zhengzhou University, Zhengzhou, Henan, China; ^2^ Department of tobacco flavor, Zhengzhou Tobacco Research Institute of CNTC, Zhengzhou, Henan, China; ^3^ Food Laboratory of Zhongyuan, Flavour Science Research Center of Zhengzhou University, Zhengzhou, Henan, China; ^4^ Medical Laboratory, General Hospital of Ningxia Medical University, Yinchuan, Ningxia, China

**Keywords:** olfactory receptors, odor molecules, sensory mechanisms, structural biology, molecular dynamics simulations

## Abstract

Olfactory receptors, classified as G-protein coupled receptors (GPCRs), have been a subject of scientific inquiry since the early 1950s. Historically, investigations into the sensory mechanisms of olfactory receptors were often confined to behavioral characteristics in model organisms or the expression of related proteins and genes. However, with the development of cryo-electron microscopy techniques, it has gradually become possible to decipher the specific structures of olfactory receptors in insects and humans. This has provided new insights into the binding mechanisms between odor molecules and olfactory receptors. Furthermore, due to the rapid advancements in related fields such as computer simulations, the prediction and exploration of odor molecule binding to olfactory receptors have been progressively achieved through molecular dynamics simulations. Through this comprehensive review, we aim to provide a thorough analysis of research related to the binding mechanisms between odor molecules and olfactory receptors from the perspectives of structural biology and molecular dynamics simulations. Finally, we will provide an outlook on the future of research in the field of olfactory receptor sensory mechanisms.

## 1 Introduction

Olfactory receptors are classified as a subset of G-protein coupled receptors (GPCRs), which are integral to the transduction of olfactory signals, the earliest accessible literature can be traced back to the 1950s of the previous century (Sviridenko, 1951; Skouby and Zilstorff-Pedersen, 1954). Early attention was devoted to the potential presence of olfactory receptors within the nasal cavities of both animals and humans, leading to a series of related investigations. Linda Buck and Richard Axel first cloned and identified the olfactory receptor GPCR gene family from rats, discovering a large gene family comprising approximately 1,000 distinct genes, which give rise to a corresponding number of olfactory receptor types. Their work illuminated how animals or humans perceive a wide array of odors, suggesting that the mechanism involves the initial binding of odorants to odor receptors located within the olfactory receptor neurons of the nasal epithelium (Buck and Axel, 1991). Following activation by odor molecules, these receptors generate electrical signals within the olfactory receptor neurons. These signals are subsequently transmitted to discrete regions within the olfactory bulb of the brain, and from there, relayed to other brain regions for further processing, ultimately leading to the perception of these odors by animals or humans. The recognition of this study with the Nobel Prize in 2004 instigated a substantial redirection of research efforts towards elucidating the functional mechanisms of olfactory receptors (Firestein, 2005). For example, in 2010, researchers directed their attention to two proteins produced within insects, or83 and or67d, which play pivotal roles in guiding mosquito odor recognition and aggressive behaviors (Carey Et Al., 2010; Wang and Anderson, 2009); In 2012, scientists at the Rowland Institute at Harvard University uncovered how the asymmetry in neurotransmitter release aids fruit flies in rapid odor recognition. Additionally, subtle differences in the excitation timing and rate of olfactory receptor neurons (ORNs) were found to variations in olfactory behavior (Gaudry et al., 2012); In 2019, researchers at Columbia University in the United States, utilizing *in situ* Hi-C technology and focusing on mice as their study subjects, revealed the extensive formation of specific trans-interactions within the three-dimensional genomic space of each olfactory neuron, these interactions serve to regulate the expression of various OR genes, thereby giving rise to the diverse combinations of olfactory receptors necessary for odor recognition (Monahan et al., 2019).

The advent of cryo-electron microscopy technology dates back to 1970s (Taylor and Glaeser, 1974), culminating in a significant milestone in late 1981 when scientists Alasdair McDowall and Jacques Dubochet reported the successful outcomes of their cryo-electron microscopy observations and, subsequently, the mastery of cryo-preservation techniques (Dubochet et al., 1981). With the onset of the 21st century, marked by remarkable advancements in computer technology and hardware capabilities, the resolution of cryo-electron microscopy has experienced remarkable enhancements. Notably, in 2013, researchers at the University of California, San Francisco, achieved near-atomic-level resolution in the visualization of membrane protein structures, heralding the commencement of a new era in protein structural analysis through the application of cryo-electron microscopy technology (Cheng, 2018; Liao et al., 2013). These pioneering strides in the field culminated in the joint recognition of Jacques Dubochet, Joachim Frank, and Richard Henderson with the Nobel Prize in Chemistry in 2017 for their substantial contributions to the development of cryo-electron microscopy technology (Dubochet, 2018). The swift progress in cryo-electron microscopy technology has concurrently ushered in novel avenues for the investigation of human olfactory receptors. In the year 2023, an unprecedented revelation emerged as the three-dimensional architecture of odorant molecules, responsible for activating olfactory receptors, was unveiled at the molecular scale (Billesbolle et al., 2023). This momentous achievement stands as a pivotal milestone in unraveling the mysteries of the sense of smell. The pioneering study illuminated the intricate binding mechanisms between odorant molecules and olfactory receptors, thereby providing unprecedented insights into the human perception of odors.

In this study, the activation mechanisms of olfactory receptors were delineated by integrating cryo-electron microscopy with molecular dynamics simulations. Olfactory receptor proteins exhibit the canonical structure of G protein-coupled receptors (GPCRs), featuring seven transmembrane domains (7TM) and encompassing three Intracellular loops (ICL) along with three Extracellular loops (ECL). Researchers have discerned that the structural alterations induced in Extracellular Loop 3 (ECL3) by the fatty acid propionate, functioning as an odorant molecule, have the capacity to trigger the activation of the human olfactory receptor OR51E2 (Billesbolle et al., 2023). This underscores the profound significance of molecular dynamics simulations as an innovative tool for unraveling the intricacies governing the interaction between small molecular ligands and receptor proteins. Molecular dynamics simulation stands as a potent and indispensable tool for delving into the intricate dynamics exhibited by biomolecules, including proteins and DNA. This technique empowers researchers to meticulously simulate the three-dimensional motions of biomolecules, facilitating the dissection of the fundamental mechanisms governing their physiological functions and the intricate interactions with potential ligands (Hollingsworth and Dror, 2018; Rydzewski and Nowak, 2017). While contemporary cryo-electron microscopy technology may still encounter limitations in deciphering the structural details of all olfactory receptor proteins, molecular dynamics simulation assumes a pivotal role in elucidating the intricate binding kinetics of odorant molecules to receptors. The combination of AlphaFold2’s 3D protein structure prediction with molecular dynamics simulations has significantly broadened their applications. This has propelled progress in deciphering molecular mechanisms, protein design, and drug development (Jumper et al., 2021a; Tunyasuvunakool et al., 2021).

**FIGURE 1:**
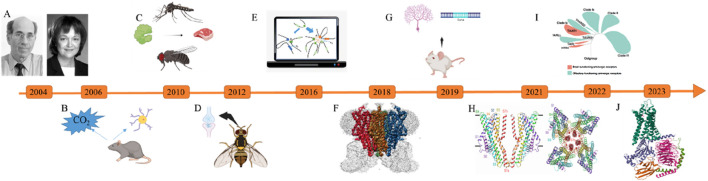
Significant research breakthroughs in olfactory receptors from 2004 to 2024. **(A)** The Nobel Prize in Physiology or Medicine (Buck and Axel, 1991); **(B)** Mice have special olfactory neurons that can sense carbon dioxide in the air (Hu et al., 2007); **(C)** Protein-Mediated Odor Recognition in Mosquitoes and *Drosophila melanogaster* (Wang and Anderson, 2009); **(D)** Asymmetric Neurotransmitter Release Facilitates Rapid Odor Recognition in Fruit Flies (Gaudry et al., 2012); **(E)** Through computational simulations, the study elucidates how olfactory neurons achieve monoallelic expression of olfactory receptors while maintaining diversity in their expression (Tian et al., 2016); **(F)** Structural Analysis of the Insect Olfactory Receptor Orco Using Cryo-Electron Microscopy (Butterwick et al., 2018); **(G)** The LDB1 Protein Governs the Expression of Olfactory Genes (Monahan et al., 2019); **(H)** Structural Analysis of the OR5 Receptor and Odor Molecule Binding Mechanisms in Machilis hrabei (Del Mármol et al., 2021); **(I)** The study elucidates the evolutionary origins, history, and co-evolutionary process of ligand recognition for a family of olfactory receptors known as trace amine-associated receptors (Guo et al., 2022); **(J)** The Inaugural Precision Three-Dimensional Structural Mapping of the Human Odor Receptor (OR51E2) Has Been Accomplished (Billesbolle et al., 2023).

The undeniable truth lies in the swift strides made within the domains of cryo-electron microscopy and computational technologies, profoundly catalyzing human endeavors in the realm of olfactory receptor research. Experimental inquiries have transcended the conventional boundaries of gene or protein functions, venturing into the intricacies of molecular mechanisms at a more microscopic scale. Table 1 and Figure 1 chronicle the pivotal research milestones pertinent to olfactory receptor investigations since 2004. This paradigm shift has transformed biological inquiry into research based on structure mechanisms. This review endeavors to accentuate the deorphaning process of olfactory receptors, along with the noteworthy advancements in elucidating olfactory receptor structures and employing molecular dynamics simulations within this field. Our aspiration is that this review will foster a deeper comprehension and foresight regarding the prospective trends in olfactory receptor research.

**TABLE 1:** Deorphanized olfactory receptor (ORs) with corresponding ligand.

Receptor name	Ligand	Reference
OR1A1	(+)-carvone (S)-(-)-citronellal helional heptanal octanal nonanal hidroxy-citronellal citral 4-decenal octanol (S)-(-)-citronellol	Mainland et al. (2015) Silva Teixeira et al. (2016)
OR1A2	(S)-(-)-citronellal helional heptanal octanal nonanal hidroxy-citronellal citral 4-decenal octanol	Silva Teixeira et al. (2016)
OR1C1	linalool	Mainland et al. (2015)
OR1D2	3-octen-2-one undecanal bourgeonal	Yasunaga et al. (2022) Kalbe et al. (2016)
OR1E3	acetophenone	Zhou et al. (2023)
OR1G1	methyl salicylate 2-undecanone ethyl isobutyrate tridecanal isoamyl acetate octopamine 9-decen-1-ol 1-nonanol ethyl isobutyrat 3-methyl-1-pentanol γ-decalactone 2-ethyl-1-hexanol	Mei et al. (2023) Topin et al. (2014) Sanz et al. (2008)
OR1L3	vanillin α-damascone	Gonzalez-Kristeller et al. (2015)
OR2A4	cyclohexyl salicylate isononyl alcohol	Tsai et al. (2016)
OR2A7	cyclohexyl salicylate α-pinene dodecanoic acid lilial octanoic acid	Tsai et al. (2016) Yasi et al. (2019)
OR2AG1	amyl butyrate	Kalbe et al. (2016)
OR2AG2	citronellol nerol cis-3-hexenol linalool geraniol α-cinnamyl alcohol phenyl ethyl alcohol phenyl propyl alcohol benzyl acetone	Duroux et al. (2020)
OR2AT4	sandalore	Edelkamp et al. (2023)
OR2A25	geranyl acetate	Mainland et al. (2015)
OR2A42	α-pinene farnesol	Yasi et al. (2019)
OR2B11	quinolone coumarin	Ben khemis and Ben Lamine (2021)
OR2B3	eugenyl acetate nerolidol β-ionone	Gonzalez-kristeller et al. (2015)
OR2C1	octanethiol heparin nonanethiol	Mainland et al. (2015) Yuan et al. (2023) Zhou et al. (2023)
OR2G2	maltyl isobutyrate cinnamaldehyde vanillin α-damascone	Gonzalez-Kristeller et al. (2015)
OR2H2	tridecanaldehyde nerol	Kim et al. (2023), Weidinger et al. (2021)
OR2J2	cis-3-hexen-1-ol 1-heptanol 1-octanol 1-nonanol 1-decanol coumarin ethyl vanillin	Mainland et al. (2015) Zhou et al. (2023)
OR2J3	cis-3-hexen-1-ol helional geranyl acetate cinnamaldehyde	Mainland et al. (2015), Mcrae et al. (2012) Kalbe et al. (2017) zhou et al. (2023)
OR2L13	1-dodecanol octanoic acid undecanal	Yasi et al. (2019)
OR2M3	3-mercapto-2-methylpentan-1-ol	Haag et al. (2019), Noe et al. (2017)
OR2M4	fructone cinnamaldehyde vanillin nerolidol α-damascone estragole cresyl methyl ether	Gonzalez-Kristeller et al. (2015)
OR2M7	Geraniol (−)-β-citronellol	Zhou et al. (2023)
OR2T4	α-pinene farnesol lilial; p-cymene undecanal	Yasi et al. (2019)
OR2T10	maltyl isobutyrate terpinyl acetate; cinnamaldehyde vanillin α-damascone	Gonzalez-Kristeller et al. (2015)
OR2T11	tert-butylthiol ethyl mercaptan	Li et al. (2016)
OR2T34	fructone; cinnamaldehyde floralozone vanillin; α-damascone jasmonyl estragole	Gonzalez-Kristeller et al. (2015)
OR2W1	cis-3-hexen-1-ol furfuryl sulfide furfuryl disulfide benzyl methyl disulfide furfuryl methyl disulfide benzyl methyl sulfide 1-phenylethanethiol benzyl mercaptan furfuryl methyl sulfide 3-phenylpropanol (+)-carvone coffee difuran allyl phenyl acetate 1-octanol helional nonanoic acid d-dimonene eugenyl acetate coumarin nonyl aldehyde octanethiol methyl	Mainland et al. (2015) Ben Khemis et al. (2023a) oh (2021)
OR2W3	tridecanaldehyde nerol	Weidinger et al. (2021), Huang et al. (2020)
OR3A1	lilial foliaver helional cyclosal tripropyleneglycolmonomethylethertrifernal methyl-phenyl-pentanal methyl-hudro-cinnamaldehyde bourgeonal methyl-cinnamaldehyde hidro-cinnamaldehyde	Silva teixeira et al. (2016)
OR3A4	2-methylisoborneol	Son et al. (2015)
OR4D1	5α-androst-16-en-3-one	Hartmann et al. (2013)
OR4D6	β-ionone galaxolide	Jaeger et al. (2013) Li et al. (2022)
OR4D9	β-ionone	Jaeger et al. (2013)
OR4E2	amyl acetate	Mainland et al. (2014)
OR4M1	asprosin	Ovali and Bozgeyik (2022)
OR4Q3	eugenol	Mainland et al. (2015)
OR5A1	β-ionone	Jaeger et al. (2013), Li et al. (2022)
OR5A2	cyclopentadec-4-en-1-one 16-hexadecanolide isomuscone 5-cyclohexadecen oxalide 11-oxahexadecanolide musk tibetene ethylene dodecanoate cervolide muscone rosamusk β-ionone	Yoshikawa et al. (2022) Jaeger et al. (2013)
OR5AC2	maltyl isobutyrate fructone eugenyl acetate manzanate vanillin α-damascone	Gonzalez-Kristeller et al. (2015)
OR5AN1	musk ketone musk xylene muscenone delta muscone xylol	Trimmer et al. (2023), Ben Khemis et al. (2021)
OR5B17	eugenyl acetate floralozone	Gonzalez-Kristeller et al. (2015)
OR5D18	eugenol isoeugenol	Zhou et al. (2023)
OR5K1	eugenol methyl trimethylthiazoline 2,3,5-trimethylpyrazine 2,5-dihydro-2,4,5-trimethylthiazoline	Mainland et al. (2015) Ben Khemis et al. (2023b) marcinek et al. (2021)
OR5M3	pentadecanal furaneol homofuraneol	Mei et al. (2023) Haag et al. (2021)
OR5P3	coumarin 1-hexanol 1-heptanol (−)-carvone (+)-carvone,Acetophenone 1-octanol celery ketone	Mainland et al. (2015), Zhou et al. (2023)
OR6A2	octanal	Orecchioni et al. (2022)
OR6M1	anthraquinone rutin	Choi et al. (2021)
OR6P1	2,5-dimethyl-3(2H)-furanone anisaldehyde	Mainland et al. (2015) Zhou et al. (2023)
OR7A5	4-hydroxy-2,5-dimethyl-3(2H)-furanone	Hartmann et al. (2013)
OR7C1	4,16-androstadien-3-one anisaldehyde	Mainland et al. (2015) Zhou et al. (2023)
OR7D4	5α-androst-16-en-3-one androstenone androstadienone	Mainland et al. (2015) choi and yoon (2021), Zhuang et al. (2009)
OR8B3	(+)-carvone	Zhou et al. (2023)
OR8D1	caramel furanone	Mainland et al. (2015)
OR8K3	(+)-menthol	Mainland et al. (2015)
OR9Q2	4-methylphenol 4-ethylphenol	Haag et al. (2023)
OR10A3	suberic acid	Kang et al. (2022)
OR10A6	3-phenyl propyl propionate citronellol nerol linalool geraniol α-cinnamyl alcohol cyclamen aldehyde lyral α-ionone phenyl ethyl alcohol phenyl propyl alcohol benzyl acetone cyclemone a nonadecane	Mainland et al. (2015), Nakanishi et al. (2023) Duroux et al. (2020)
OR10G3	vanillin ethyl vanillin	Mainland et al. (2015), Zhou et al. (2023)
OR10G4	guaiacol vanillin ethyl vanillin quinoline coumarin	Mainland et al. (2015), Mainland et al. (2014) Ben Khemis and Ben Lamine (2021)
OR10G7	eugenol acetophenone	Mainland et al. (2015) Tham et al. (2019)
OR10G9	ethyl vanillin	Zhou et al. (2023)
OR10H1	sandranol	Weber et al. (2018)
OR10J5	lyral	Mainland et al. (2015), Curtis et al. (2023), Ben Khemis et al. (2022)
OR10S1	1-dodecanol 1-octanol geraniol heptanoic acid lilial nonanal octanoic acid	Yasi et al. (2019)
OR11A1	2-ethyl fenchol	Mainland et al. (2015)
OR11H4	phenyl ethyl alcohol phenyl propyl alcohol isovaleric acid	Duroux et al. (2020) Zhou et al. (2023)
OR11H6	isovaleric acid	Zhou et al. (2023)
OR11H7P	isovaleric acid	Menashe et al. (2007)
OR51A7	β-ionone	Murayama et al. (2023)
OR51B4	troenan	Weber et al. (2017)
OR51B5	isononyl alcohol dodecanoic acid farnesol	Manteniotis et al. (2016) Yasi et al. (2019)
OR51E1	isovaleric acid butyrate cyclobutanecarboxylic acid 2-methylbutyric acid nonanoic acid decanoic acid	Mainland et al. (2015) Xu and Pluznick (2022) Bushdid et al. (2018) Massberg et al. (2016)
OR51E2	β-ionone propionate acetate	Murayama et al. (2023), Xu et al. (2022) Billesbolle et al. (2023)
OR51L1	allyl phenyl acetate hexanoic acid allyl phenyl acetate	Mainland et al. (2015) Zhou et al. (2023)
OR51S1	geosmin	Son et al. (2015)
OR52D1	ethyl heptanoate methyl octanoate 1-nonanol 2-nonanol 3-nonanone 3-octanone	Zhou et al. (2023)
OR52H1	2,3-butandione	Zeng et al. (2023)
OR52J3	2,3-butandione	Zeng et al. (2023)
OR56A1	undecanal	Zhou et al. (2023)
OR56A4	decyl adehyde undecanal	Zhou et al. (2023)
OR56A5	undecanal	Zhou et al. (2023)

## 2 Olfactory receptor deorphanization and current research status

### 2.1 The importance of olfactory receptor deorphaning

Within the realm of scientific exploration, olfactory receptors constitute a prominent segment of the G-protein-coupled receptor (GPCR) superfamily, ranking among the most extensive protein families in the mammalian world (Peterlin et al., 2014). These receptors shoulder the responsibility of detecting and discerning a myriad of odorant molecules. Nevertheless, the current landscape reveals that more than 80% of olfactory receptors remain enigmatic orphan receptors while their ligands shrouded in mystery (March et al., 2015).

The pursuit of natural ligands for orphan olfactory receptors stands as a substantial domain of investigation. On one front, deorphaning empowers us with insights into the mechanisms through which the olfactory system deciphers and processes specific aromas (Peterlin et al., 2014; Reisert and Restrepo, 2009). Through the alignment of receptors with their corresponding ligands, we gain profound understanding of the molecular underpinnings and binding dynamics governing odor perception, ultimately unveiling the enigma of human sensory mechanisms. On another front, the deorphaning of olfactory receptors bears significant practical applications. For instance, within the realms of fragrance and food industries, a meticulous grasp of interactions among odor receptors can engender the creation of novel scents, particularly serving as substitutes for food additives in the evolving landscape of artificial food production (Zeng et al., 2023).

A compelling dataset underscores that in 2015, among the nearly 400 complete human olfactory receptors (ORs), only 49 ligands have been published (Mainland et al., 2015). Nevertheless, during the same year, an alternative dataset revealed that the count of human olfactory receptors that had been successfully deorphaned was also a modest 57 (March et al., 2015). At present, based on a comprehensive review of the available literature, the cumulative count of orphan olfactory receptors is estimated to exceed eighty (Table 1).

### 2.2 The research status of olfactory receptors and odor molecule ligands

Within the scientific literature, a significant corpus of research has been devoted to the pursuit of ligands for odorant molecules that trigger the activation of their respective olfactory receptors. A noteworthy development emerged as early as 2015 when a literature introduced a high-throughput screening technique tailored for olfactory receptors, effectively identifying agonists for 27 distinct odor receptors (Mainland et al., 2015). Notably, during that period, 18 of these olfactory receptors remained orphan receptors.

In 2016, Dietmar Krautwurst and his research team made a significant breakthrough by uncovering 14 novel crucial food odorant agonists for OR1A1 and 18 for OR2W1, respectively (Geithe et al., 2017). This study use dual-screening strategy, entailed the comprehensive test of a diverse spectrum of food odorant molecules with individual ORs. Subsequently, a single odor compound was tested the complete human OR, leading to the effective revelation of the intricate olfactory attributes associated with odorant molecules.

Moreover, recent years have witnessed a substantial body of research dedicated to the deorphaning of individual olfactory receptors. Pyrazine compounds, recognized as odorant molecules contributing to food enhancement, have undergone thorough investigation, leading to the revelation that OR5K1 stands as the exclusive receptor responsive to pyrazine compounds. Interestingly, homologous receptors of OR5K1 in mice have exhibited analogous activation functions (Marcinek et al., 2021). In parallel, furaneol and sotolone, essential flavoring furanones, have also undergone scrutiny. The outcomes have illuminated their distinct abilities to selectively activate the human olfactory receptors OR5M3 and OR8D1, respectively. Notably, both odorant molecules exhibited conspicuous concentration-dependent activation profiles throughout the investigation (Haag et al., 2021). Another illustrative instance pertains to 4-methylphenol, distinguished by its odor reminiscent of a stable’s fecal notes. It was discerned that OR9Q2 manifested an elevated response to this specific odorant molecule, unequivocally establishing OR9Q2 as the primary sensor for a spectrum of aromas, encompassing food odors, foul scents, and the chemical pheromone 4-methylpheno (Haag et al., 2023).

In the natural world, odorant molecules are frequently encountered in complex mixtures rather than in isolation. Consequently, the interactions among various odorant molecules remain largely uncharted. These interactions can yield diverse outcomes, ranging from the inhibition or potentiation of specific odor perceptions to the emergence of entirely novel and unpredictable olfactory sensations (Poupon et al., 2018; Thomas-Danguin et al., 2014). For example, the response of all odorant receptors to a moderate concentration of the malodorous compound indole, reminiscent of fecal odor, is effectively suppressed by the presence of a high concentration of the floral odorant α-ionone (Breheny et al., 2020). When present in mixtures with a high concentration of whisky lactone, olfactory receptors responsible for detecting the fruity aroma of isoamyl acetate undergo inhibition (Chaput et al., 2012). Additionally, it has been observed that undecanal can effectively diminish the sensitivity of olfactory receptors to bourgeonal (Brodin et al., 2009; De march et al., 2020).

Within well-known complex odor amalgamations, cigarette smoke stands as a prevalent and intricate olfactory ensemble, consisting of more than 400 distinct odorant molecules (Cortese et al., 2015). Experimental observations in mice have unveiled that the scent of cigarette smoke activates a remarkable response from 144 olfactory receptors (ORs) and 3 trace amine-associated receptors (TAARs). Moreover, sensory evaluation studies, have underscored the pivotal role of 1-pentanethiol as a significant constituent contributing to the distinctive aroma profile of synthetic cigarette smoke (Mcclintock et al., 2020).

### 2.3 The limitations of research on olfactory receptors

While there has been a surge in research on olfactory receptors and their deorphaning in recent years, the exploration of binding mechanisms between odorant molecules and olfactory receptors has remained relatively limited. This research gap becomes particularly evident when considering molecular ligands that exhibit stereoisomerism, such as chiral isomers or functional group isomers. Fundamental questions about the varying activation abilities of these isomers, the migration of optimal binding sites, and the binding conformations between odorant molecule ligands and olfactory receptors have largely remained unaddressed (Behrens et al., 2018). Additionally, comprehending the intermolecular forces at play between functional groups of ligands and amino acid residues in olfactory receptors has posed significant challenges.

Furthermore, olfactory receptors are dynamic proteins, undergoing conformational changes between active and inactive states (García-Nafría and Tate, 2021). Investigating these dynamic changes at the microscale level presents formidable challenges in deorphaning studies.

In light of these complexities, the growing interest in olfactory receptors underscores the imperative need for further research into the intricate details of odorant molecule and olfactory receptor interactions, particularly when confronted with structural isomerism and conformational dynamics.

## 3 Olfactory receptor and structural biology

### 3.1 Olfactory receptor and cryo-electron microscopy technology

Structural biology involves elucidating the three-dimensional arrangements of biological macromolecules at the atomic level and employing these structures to decipher the chemical underpinnings of their biological functions (Moore, 2017). In the early 20th century, X-ray crystallography gained widespread popularity as a technique for studying the structures of proteins and nucleic acids (Gaubert et al., 2020). By the mid-20th century, nuclear magnetic resonance (NMR) and protein crystallography emerged as an equally potent tool for elucidating the structures of biological molecules (Koehler leman and Künze, 2023).

Recent years, the advancement of cryo-electron microscopy technology has brought about a revolutionary change in the field of structural biology, offering scientists a pioneering instrument. This technique empowers researchers to scrutinize protein structures, including those of olfactory receptors, at remarkably high resolutions (Yip et al., 2020; Danev et al., 2019). With cryo-electron microscopy, investigators can unveil the intricate three-dimensional architecture of olfactory receptors, thereby facilitating a more profound comprehension of their mechanisms for recognizing odorant molecules.

In 2018, scientists from Rockefeller University’s Ruta Lab in the United States achieved a groundbreaking milestone by unveiling the single-particle cryo-electron microscopy structure of the odorant co-receptor Orco in a parasitic wasp, boasting an impressive resolution of approximately 3.5 Å (Butterwick et al., 2018). Insect odorant receptors diverge from their mammalian counterparts in their classification as ligand-gated ion channels rather than G-protein-coupled receptors (GPCRs). These receptors assemble into heteromeric ion channel complexes, consisting of ORs and the exceptionally conserved co-receptor, Orco. This ion channel functions akin to a conduit, permitting the flow of charged particles solely when the receptor encounters its intended odorant molecule, thereby initiating the activation of olfactory sensory cells.

In 2021, a separate research endeavor conducted by the Ruta Lab brought to light the cryo-electron microscopy structure of an odorant receptor found in Machilis hrabei, a species of stonefly. These terrestrial insects, whose genome has been recently sequenced, possess only five odorant receptors. The research team delved deeper into the examination of the binding sites and mechanisms of two chemically distinct molecules with the OR5 receptor. Furthermore, they conducted a comparative analysis of the structural alterations in OR5 induced by the binding of various odorant molecules. The study demonstrates that upon binding with eugenol, the receptor’s structure exhibits pore dilation, providing a channel for ion flow. Additionally, it was found that amino acids connected to the “pocket” do not form strong, selective chemical bonds with the odorant, but rather form weak bonds (Del mármol et al., 2021).

As of March 2023, an article published in Nature has, for the very first time, elucidated the enigmas shrouding the structure of human olfactory receptors. This study provides intricate insights into an olfactory receptor denoted as OR51E2, elucidating its ability to “discern” the aroma of cheese by engaging in precise molecular interactions, thus initiating receptor activation (Billesbolle et al., 2023).

### 3.2 Human olfactory receptor structures and the mechanisms olfactory sensation

**FIGURE 2:**
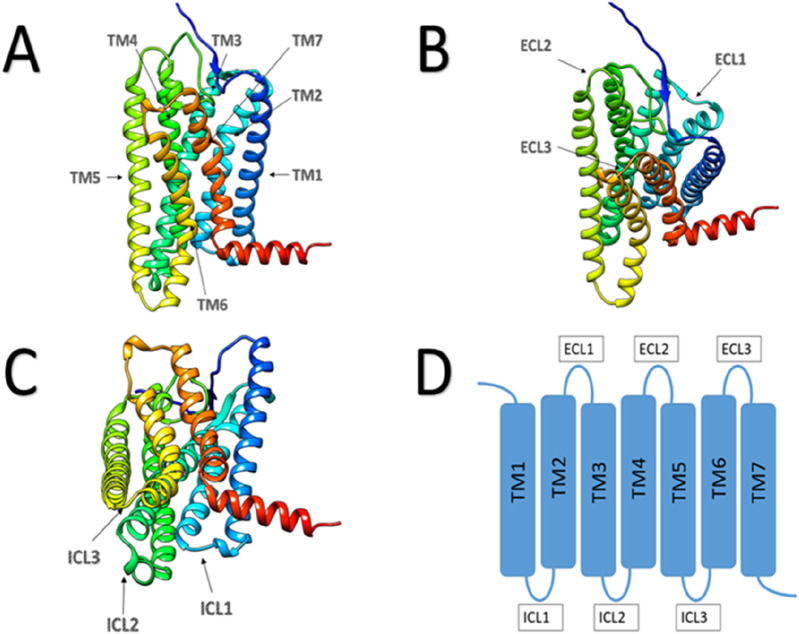
Schematic diagram of GPCR protein structure. **(A)** Main view of GPCR protein, with arrows indicating the seven transmembrane regions. **(B)** Top view of GPCR protein, with arrows pointing to three extracellular loops. **(C)** Bottom view of GPCR protein, with arrows pointing to three intracellular loops. **(D)** Plan view of GPCR protein structure.

We already know, human odorant receptors are G-protein-coupled receptors (GPCRs) (Gaillard et al., 2004). A common feature of these receptors is that they all possess seven transmembrane α-helices in their three-dimensional structure (Figure 2).

Structural determination of human odorant receptor proteins poses a significant challenge for several reasons. Firstly, the sheer diversity of odorant receptors means that each possesses its distinct structural traits; secondly, the relatively low expression levels of these receptors in sensory cells introduce technical obstacles when trying to elucidate their protein structures through cryo-electron microscopy techniques; moreover, odorant receptors exhibit dynamic properties, with their conformations capable of altering upon binding different odorant molecules (Ikegami et al., 2020; Saito et al., 2004; Cook et al., 2009; Katada et al., 2004). This intricacy adds an additional layer of complexity to structural analysis under various conditions.

To tackle these formidable challenges, researchers opted to investigate the human olfactory receptor OR51E2 for specific reasons. Their choice was motivated by the receptor’s expression not only in olfactory nerve cells but also in non-olfactory organs such as the prostate. This dual expression pattern suggested that it would be more feasible to express the receptor in heterologous systems, making it easier to produce sufficient quantities of the protein. Previous studies had already demonstrated that this receptor could bind to and elicit responses from water-soluble short-chain fatty acid odorant molecules, particularly propionic acid (Saito et al., 2009). Through these strategic decisions, researchers adeptly navigated the challenges presented by the typically low expression levels of most odorant receptors, the low solubility of many volatile odorants, and the inherent instability of purified olfactory receptor proteins.

Structural examination of OR51E2 unveiled a fascinating mechanism: the receptor protein effectively entraps the odorant molecule propionic acid within a compact, enclosed binding pocket. Within this minute enclosure, propionic acid forms two types of interactions with OR51E2: polar interactions involving hydrogen and ionic bonds, as well as non-specific hydrophobic interactions (Billesbolle et al., 2023). Consequently, the way OR51E2 binds to odorant molecules differs significantly from that of insect odorant-gated ion channels, implying a heightened level of selectivity.

This discovery also elucidates why OR51E2 exclusively binds to short-chain fatty acids (Pluznick et al., 2013; Pronin and Slepak, 2021). The binding pocket’s limited volume, measuring 31 Å^3^, accommodates short-chain fatty acids like acetic and propionic acid, while effectively preventing the binding of longer fatty acid chains. Mutation of phenylalanine and leucine residues adjacent to the fatty acid propionate to the smaller alanine residue enlarges the binding pocket, facilitating the activation of OR51E2 by long-chain fatty acids. Thus, this revelation underscores that the volume of the binding pocket plays a pivotal role in determining the receptor’s selectivity for odorant molecules. This study has undeniably offered groundbreaking insights into the atomic-level structure and mechanisms governing OR51E2 function.

### 3.3 Limitations of olfactory receptors in the structural biology

Certainly, cryo-electron microscopy technology has ventured into the microscopic realm of olfactory receptors and sensory mechanisms, reshaping traditional biological inquiries into chemical explorations. Nevertheless, the comprehensive structural characterization of numerous human olfactory receptor proteins remains an arduous undertaking today. This challenge stems from multiple factors, including the limited expression levels of olfactory receptor proteins, their structural heterogeneity, and their dynamic conformational alterations. Furthermore, the intricacies of the interactions between odorant molecules and olfactory receptors should not be underestimated, encompassing hydrophobic interactions, hydrogen bonds, ionic bonds, and more. Consequently, it is essential to employ a diverse array of approaches to scrutinize these multifaceted binding mechanisms.

## 4 Olfactory receptor and molecular dynamic simulation

### 4.1 Molecular dynamic simulation (MD) and AlphaFold

Over the past few decades, the determination of protein structures has predominantly relied on various experimental techniques, including early methodologies such as X-ray crystallography and nuclear magnetic resonance (NMR) (Faraggi et al., 2017), as well as more recent innovations like cryo-electron microscopy (Yip et al., 2020). Nevertheless, these approaches are often laborious and time-intensive, involving extensive trial and error. Unraveling the structure of a single protein can span several years and necessitates the use of specialized equipment that comes with a multi-million-dollar price tag.

In 2020, the emergence of the AlphaFold2 (AF2) program marked a pivotal moment, demonstrating remarkable accuracy in predicting the three-dimensional structures of a substantial number of proteins (Bryant et al., 2022). Furthermore, in July 2021, the AlphaFold2 system unveiled a database containing protein structures it had predicted (https://alphafold.ebi.ac.uk/) (Jones and Thornton, 2022). On 15 May 2024, the Google DeepMind team in collaboration with Isomorphic Labs reported a groundbreaking model, AlphaFold 3 (Abramson et al., 2024), which is capable of predicting the complex structures composed of DNA, small molecules, ions, and proteins, as well as forecasting the structures and interactions of all biomolecules. The advent of AlphaFold 3 marks a significant leap forward in the field of structural biology, with profound implications for understanding the molecular basis of life. It is foreseen that AI-driven protein structure prediction will continue to achieve rapid breakthroughs over the next 2–5 years, holding significant implications for the field of drug discovery in the foreseeable future.

In the realm of computational chemistry and biophysics, Molecular Dynamics Simulation (MD) stands as a ubiquitous computational tool revered for its prowess in probing the intricate dynamics of atoms and molecules, tracing their evolution over time (Hildebrand et al., 2019). MD simulations find widespread applications across various domains, encompassing biophysics (Collier et al., 2020) (for the study of the structure and function of proteins and biomolecules), materials science (Xie et al., 2022) (for the exploration of material properties and phase transitions), and chemistry (Park, 2016) (for the examination of chemical reaction kinetics), among others. Equally paramount is MD’s role in the exploration of olfaction mechanisms, where it assumes a central position in unraveling the nuanced interplay between olfactory receptors and the molecules that tantalize our senses. Moreover, it is noteworthy that an intimate nexus exists between MD and AlphaFold. Prior to the advent of AF2, researchers predominantly relied upon homology modeling to construct the three-dimensional structures of olfactory receptor proteins, complemented by virtual screening techniques to unearth ligands that would unlock the mysteries of these receptors (De march et al., 2018; Cong et al., 2022). However, it has come to light in comparative studies that there exist disparities between the olfactory receptor protein structures derived from homology modeling and those obtained through AF2. For example, OR5K1, a major difference between HM and AF2 models is in the Extracellular Loop 2 (ECL2) folding. The ECL2 predicted by AF2 seems unique and was found to be rather stable in MD simulations (Nicoli et al., 2023).

Therefore, the introduction of AlphaFold has greatly enhanced the precision of predicting protein structures, even in scenarios where there is no apparent homologous or low homologous protein available. This feature stands out as superior to the homology modeling approach that relies on known protein structures as templates.

### 4.2 MD and olfactory mechanisms

MD can be employed in various aspects of olfactory receptor mechanism studies. For instance, MD can replicate the structural dynamics of olfactory receptors, encompassing transitions between different conformations. This aids in comprehending the structural characteristics of receptors in different states, such as active and inactive states. Additionally, it can elucidate the binding modes and affinities between olfactory receptors and odorant molecules (ligands), shedding light on how receptors identify and interact with different odor molecules. Furthermore, MD can simulate the activation process of olfactory receptors, including their interaction with G proteins and the intricate details of signal transduction mechanisms. This contributes to revealing how receptors transduce external signals into intracellular biological responses. Lastly, by leveraging the known features of ligands and olfactory receptors, it becomes possible to predict novel antagonists and agonists.

GPCRs serve as the molecular conduits for transmitting chemical signals, orchestrating an intricate ballet that transitions between active and inactive states upon ligand binding, thereby bridging the chasm from extracellular to intracellular domains (Weis and Kobilka, 2018; Alhadeff et al., 2018). Yet, it is notable that AF3, in its current form, falls short by primarily predicting a solitary state and exhibiting a propensity to favor either active or inactive conformations contingent upon the specific GPCR class under scrutiny (Jumper et al., 2021b; Kinch et al., 2021). MD empowers researchers to transcend the constraints of AF3’s singular state predictions and venture into the modeling of both active and inactive states. In doing so, MD gracefully captures the intricate and pivotal structural transformations that transpire within receptors during the delicate interplay of activation and deactivation (Heo and Feig, 2022).

The investigation into how odor molecules engage with olfactory receptors stands as a notable focal point within the domain of MD research. The scientific literature has delved into the mechanisms governing the interactions between 18 caramel-like odorants and receptors, unveiling a distinct preference for odor molecules to establish bonds with the transmembrane regions TM-3, TM-5, and TM-6 of olfactory receptors (Katada et al., 2005). Through meticulous analysis and computational simulations of caramel-like odors, it has come to light that hydrogen bonding and π-π stacking assume pivotal roles in conferring stability upon these aromatic compounds. Incorporating the paradigm of molecular field-based similarity analysis has yielded two noteworthy ligands: 4-hydroxy-5-methylfuran-3(2H)-one and methylglyoxal. These compounds exhibit a pronounced affinity for binding to receptors OR1G1 and OR52H1, respectively, thereby eliciting sensory perceptions akin to the enticing aroma of caramel (Zeng et al., 2023).

**FIGURE 3:**
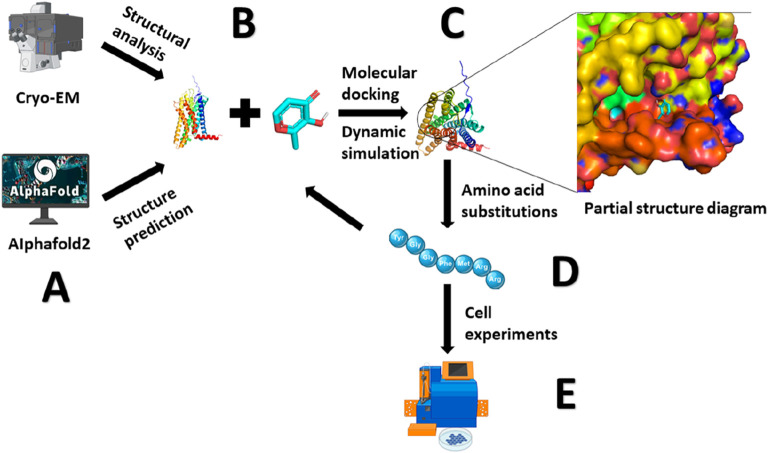
Methods for Studying the Olfactory Receptor - Odorant Binding Mechanism. **(A)** Resolving protein structures through cryo-electron microscopy (Cryo-EM) or predicting protein conformations using AlphaFold2. **(B)** Using molecular docking and molecular dynamics simulations to bind odorants with olfactory receptors. **(C)** Analyzing the interactions forces between protein and ligand. **(D)** Identifying crucial amino acid residues, inducing mutations, molecular docking and molecular dynamics simulations, and further analyzing the binding mechanism. **(E)** Cellular experiments are conducted to detect the activation of olfactory receptors, validating the proposed mechanism.

In our previous discussion, we highlighted that olfactory receptors are part of the G protein-coupled receptor family, characterized by their transmembrane structure comprising seven α-helices. In addition to these transmembrane regions, olfactory receptors feature three extracellular loops (ECLs) and three intracellular loops (ICLs) (Venkatakrishnan et al., 2013). A specific study has illuminated the pivotal role played by ECL2 in shaping and regulating the volume of the odorant-binding pocket. ECL2 also maintains the pocket’s hydrophobic properties and serves as a gatekeeper for odorant binding (Yu et al., 2022). This underscores the paramount significance of Olfactory Receptor ECL2 in influencing both the diversity and specificity of olfactory receptor responses. In the investigation of OR51E2, conformational alterations within ECL3 play an equally pivotal role in the activation of OR51E2. The authors hypothesize that ECL3 in olfactory receptors plays a role in stabilizing odorants, which is conducive to the further activation of olfactory receptors by the odorants. This stabilization is essential for the diverse activation of olfactory receptors necessary for odor recognition (Billesbolle et al., 2023) (Figure 3).

To aid in deorphanization of ORs, Jérôme Golebiowski and his team have developed a Protein Chemistry Metric (PCM) model based on OR sequence similarity and the physicochemical characteristics of ligands. This model employs supervised machine learning to predict odor responses by ORs. Starting from a dataset of OR-odorant pairs and considering the surrounding 60 residues of the binding pocket, this model forecasts changes in OR responses to odors. Remarkably, it accomplishes these predictions with less than 20% of the residue sequence. The model achieves an impressive hit rate of 58%, uncovering 64 novel odorant-OR pairs (Cong et al., 2022). Another study have revealed that, through the utilization of molecular docking and virtual screening techniques, novel antagonists or agonists for mOR256-3 have been successfully identified. Remarkably, these findings have been substantiated by cell-based assays, demonstrating an impressive 70% success rate (Yu et al., 2022).

### 4.3 Prospects of olfactory receptors in the field of MD

While computational simulation techniques have undeniably facilitated the investigation of olfactory mechanisms, the exploration of sensory processes associated with olfactory receptors is a relatively nascent field, spanning just over two decades (Coppola, 2022). Numerous pressing inquiries beckon researchers across the globe to delve deeper into this intriguing domain.

To begin, it is crucial to acknowledge that receptor protein activation constitutes a dynamic journey. Hence, the thorough examination of structural dynamics within olfactory receptors, encompassing the transitions between diverse conformations, assumes paramount significance. This endeavor serves as a foundational pillar for achieving a more comprehensive comprehension of their functionality and activation mechanisms. Furthermore, a more exhaustive exploration into the intricate interplay between olfactory receptors and odor molecules is imperative. This includes a meticulous scrutiny of binding modes and affinities, for it is within these specifics that the enigma of odor recognition truly lies. Lastly, leveraging the power of MD simulations to anticipate interactions between olfactory receptors and novel compounds stands as a linchpin. This pursuit holds immense potential in unearthing new pharmaceutical agents or aromatic compounds, thus advancing the frontiers of the pharmaceutical and food industries.

In summation, while MD have eased the path of exploration, the realm of olfactory receptor-based sensory mechanisms remains in its infancy. It is a realm ripe with myriad questions, awaiting the diligent investigations of researchers worldwide.

## 5 Summary and outlook

In conclusion, the research prospects pertaining to olfactory receptors are replete with promise. This article has provided an overview of recent investigations into the deorphanization of olfactory receptors, delved into the advancements within the field of structural biology, and explored pertinent findings from the domain of molecular dynamics. Nevertheless, the overarching objective within each of these domains remains the clarification of the binding mechanism governing the interaction between odor molecules and olfactory receptors.

Moreover, as cryo-electron microscopy technology continues to advance, it is likely to surmount challenges such as the low expression and instability of olfactory receptor proteins. This opens the door to a future where the structures of numerous human olfactory receptor proteins will be systematically elucidated. The amalgamation of these structural revelations with MD will undoubtedly propel our comprehension of the mechanisms underpinning olfactory perception. This, in turn, holds the promise of catalyzing fresh innovations and opportunities across an array of domains, encompassing pharmaceuticals, food science, and the fragrance industry.
